# Evaluating a Video-Based, Personalized Webpage in Genitourinary Oncology Clinical Trials: A Phase 2 Randomized Trial

**DOI:** 10.2196/12044

**Published:** 2019-05-02

**Authors:** Rana McKay, Hannah Mills, Lillian Werner, Atish Choudhury, Toni Choueiri, Susanna Jacobus, Amanda Pace, Laura Polacek, Mark Pomerantz, Judith Prisby, Christopher Sweeney, Meghara Walsh, Mary-Ellen Taplin

**Affiliations:** 1 University of California San Diego La Jolla, CA United States; 2 Department of Medical Oncology Dana-Farber Cancer Institute Boston, MA United States

**Keywords:** cancer, prostatic neoplasms, kidney neoplasms, clinical trial, instructional films and videos, education

## Abstract

**Background:**

The pace of drug discovery and approvals has led to expanding treatments for cancer patients. Although extensive research exists regarding barriers to enrollment in oncology clinical trials, there are limited studies evaluating processes to optimize patient education, oral anticancer therapy administration, and adherence for patients enrolled in clinical trials. In this study, we assess the feasibility of a video-based, personalized webpage for patients enrolled in genitourinary oncology clinical trials involving 1 or more oral anticancer therapy.

**Objective:**

The primary objective of this trial was to assess the differences in the number of patient-initiated violations in the intervention arm compared with a control arm over 4 treatment cycles. Secondary objectives included patient satisfaction, frequently asked questions by patients on the intervention arm, patient-initiated calls to study team members, and patient-reported stress levels.

**Methods:**

Eligible patients enrolling on a therapeutic clinical trial for a genitourinary malignancy were randomized 2:1 to the intervention arm or control arm. Patients randomized to the intervention arm received access to a video-based, personalized webpage, which included videos of patients’ own clinic encounters with their providers, instructional videos on medication administration and side effects, and electronic versions of educational documents.

**Results:**

A total of 99 patients were enrolled (89 were evaluable; 66 completed 4 cycles). In total, 71% (40/56) of patients in the intervention arm had 1 or more patient-initiated violation compared with 70% (23/33) in the control arm. There was no difference in the total number of violations across 4 cycles between the 2 arms (estimate=−0.0939, 95% CI−0.6295 to 0.4418, *P* value=.73). Median baseline satisfaction scores for the intervention and control arms were 72 and 73, respectively, indicating high levels of patient satisfaction in both arms. Median baseline patient-reported stress levels were 10 and 13 for the intervention and control arms, respectively, indicating low stress levels in both arms at baseline.

**Conclusions:**

This study is among the first to evaluate a video-based, personalized webpage that provides patients with educational videos and video recordings of clinical trial appointments. Despite not meeting the primary endpoint of reduced patient-initiated violations, this study demonstrates the feasibility of a video-based, personalized webpage in clinical trials. Future research assessing this tool might be better suited for realms outside of clinical trials and might consider the use of an endpoint that assesses patient-reported outcomes directly. A major limitation of this study was the lack of prior data for estimating the null hypothesis in this population.

## Introduction

### Background

Over the past several years, the Food and Drug Administration (FDA) has approved several new therapies for the treatment of genitourinary malignancies [1], and this is largely because of patient enrollment in clinical trials [2]. Between June of 2017 and May of 2018, the FDA approved 58 oncology drugs and granted new indications for previously approved drugs, 6 of which were indicated for genitourinary malignancies [3]. Improvement of patient outcomes hinges on well-designed clinical trials [4]. Therefore, it is essential for investigators to optimize clinical trial processes and develop strategies to improve the patient experience on therapeutic clinical trials. Researchers have identified several oncology clinical trial obstacles, which include low patient enrollment, underrepresentation of minorities [5], and data collection burden on the clinical trials system [6].

Given that approximately 25% to 30% of the oncology drug pipeline involves oral anticancer therapy [7], researchers can anticipate new and evolving barriers to oncology clinical trial conduct. Self-administered oral therapies create the added responsibility of ensuring out-of-hospital drug dosing and monitoring. The transition to more oral anticancer therapy has resulted in increased challenges related to patient adherence to prescribed treatment regimens [8]. Adherence is defined as the degree to which patients follow recommendations for day-to-day treatment with respect to the timing, dosing, and frequency of oral anticancer therapy [9]. Nonadherence can result from improper timing of doses, missed doses, incorrect amount, and erratic dosing schedules. Nonadherence has been shown to affect treatment efficacy, leading to worsened survival, resistance, and treatment failure [10-12].

Adherence interventions for patients in clinical trials have typically focused on providing oral anticancer therapy education at the time of clinical trial informed consent or initial investigational drug prescription. Experience demonstrates that trials involving oral anticancer therapy necessitate additional support for patients to optimally manage treatment outside the secure and supportive clinic environment [13].

Before the rise of oral chemotherapy, patients typically received their treatments in the clinic where intravenous drugs could be administered in a safe, protected environment. Now, patients can take their oral chemotherapy at home without direct observation. The level to which adherence is an issue in oncology clinical trials is not clearly defined, and the degree of additional support patients require to properly self-administer oral anticancer therapy has not been quantified. It is known, however, that the strict requirements of a clinical trial (eg, frequent clinic visits, pharmacokinetic blood draws, and medication diary completion) can cause patients to feel overwhelmed and confused. Research has shown that patients can have misconceptions about aspects of research, such as the risk of side effects, trial aims, and the likelihood of personal benefit [14].

### Prior Research

Research looking at the use of Web-based interventions and video content to improve oral medication adherence remains largely untapped, but data do suggest that Web-based interventions can effectively increase medication adherence among chronically ill patients, patients undergoing smoking cessation, patients initiating HIV treatment, and in other clinical scenarios [15-17]. Research has also shown that patients, with their increasing aptitude for technology, benefit from innovative approaches that go beyond the written informed consent document, such as interactive Web-based tools that can be used to engage patients and improve understanding of their disease and treatment [18-21]. A systematic review and meta-analysis of studies assessing Web-based interventions compared with usual care or other decision aids within the realm of screening for prostate cancer found that of the 4 studies that used knowledge as an outcome, patients assigned to the Web-based intervention group had higher average knowledge scores than those assigned to usual care [22]. Of the 2 studies that compared Web-based interventions with video decision aids, patients’ average knowledge was higher in the group of patients using video.

Phase 3 studies have shown that video content that is tailored to the cancer patient compared with standardized text can effectively improve patient knowledge related to clinical trials and reduce attitudinal barriers [18]. Furthermore, video content has been shown not only to increase knowledge but also to increase patient satisfaction as well [23,24]. Advantages of video and Web-based modes of communication are that they can be stored, shared, and accessed repeatedly by patients and their families. On the basis of current research implications and the evidence supporting video and Web-based interventions to improve knowledge, medication adherence, stress, and satisfaction, we believe creative technology and personalization of communication will improve the patient experience and reinforce the patient care plan.

### This Study

In this randomized phase 2 study, we assessed the use of a video-based, personalized webpage via the information-sharing platform, Postwire, to help patients navigate their participation in genitourinary oncology clinical trials involving 1 or more oral anticancer therapy. We used a novel and objective endpoint, patient-initiated protocol violations, for this study to quantitatively evaluate whether a personalized, video-based webpage might help clinical trial patients better adhere to the strict clinical trial guidelines. Patient-initiated protocol violations can be objectively measured and reflect patient misunderstanding regarding medication administration and other protocol procedures. We hypothesized that patients receiving the video-based intervention would have less patient-initiated protocol violations given improved understanding about mediation administration and trial procedures, which would be provided through the video-based, personalized webpage compared with a control arm. In addition, we hypothesized improved patient satisfaction and reduced stress.

## Methods

### Study Design

This was a randomized, phase 2 clinical trial assessing a video-based, personalized webpage as a complement to standard patient education for clinical trial patients. The institutional review board (IRB) at the Dana-Farber Cancer Institute (DFCI) approved this intervention and determined that this randomized, phase 2 trial did not meet the requirements for registration to a WHO-accredited trial registry as it was an ancillary, noninvasive trial.

The Postwire application was chosen as it is Health Insurance Portability and Accountability Act (HIPAA)–compliant and uses secure sockets layer technology to prevent hacking and ensure privacy. All patients provided written informed consent. This study was pilot-tested before the initiation of patient recruitment and enrollment to optimize workflow and minimize technology malfunctions. A safety run-in of 9 weeks took place to carefully monitor patients and protocol procedures, and adjustments were made as necessary to optimize workflow and study conduct.

The study consisted of 2 arms: the video-based intervention arm and the control arm. Patients were randomized 2:1 to the intervention arm or control arm and were stratified by therapeutic or *parent* clinical trial protocol type (targeted, hormone, or combination therapy). Patients randomized to the control arm received standard clinical trial oral anticancer therapy education and care without a video-based personalized webpage. Patients enrolled in the intervention arm had access to a video-based personalized webpage in addition to standard of care educational materials.

### Intervention Arm

Patients assigned to the intervention arm were introduced to the Postwire interface prior to cycle 1 day 1. A designated clinical research coordinator used a tablet to show the patient how to navigate the personalized webpage using a template page. The study team obtained the patient’s email address, and within 24 hours of the patient’s cycle 1 day 1 clinic visit, a secure email containing a link to the patient’s personalized webpage was sent. The next 3 clinic visits with the patient’s oncology provider were recorded in real time and uploaded to the patient’s personalized webpage. This webpage included videos of the patient’s own clinic encounters, their parent clinical trial informed consent, emergency contact information, oral anticancer therapy dosing guidelines, and medication diaries. When new or revised content was uploaded to a patient’s personalized webpage, a notification email was sent. In addition to the video recordings of patient’s encounters with their providers, the webpage was equipped with templated videos of the patients’ own nurses describing how to self-administer oral anticancer therapy (Multimedia Appendix 1), what side effects to expect versus side effects requiring an emergency room visit or a call to the study team, and how to complete study-required documents, such as a medication diary. Patients had the ability to share their webpage access with caregivers (referred to as *patient designees*).

The personalized webpage was available to patients on the intervention arm until 6 full cycles had been completed. Once data collection was complete, the webpage was deleted. If a patient withdrew from the parent, therapeutic clinical trial, or this ancillary trial, the webpage was deactivated. All patients on the intervention arm received paper-based instructional documents and were encouraged to contact the study team with any questions. This intervention was a complement to standard practice rather than a replacement. Patients randomized to the control arm received the same educational content in paper form and were educated by their treating oncologists and care teams according to standard practice.

### Study Calendar

This ancillary study’s treatment cycles matched the patient’s parent clinical trial treatment cycles, which ranged from 21 to 42 days except for 1 trial which had 90-day treatment cycles after the first 28 days. Patients remained on the intervention for 6 cycles (4 cycles of the intervention and 2 cycles of follow-up), withdrawal of consent, or until meeting parent trial discontinuation criteria.

### Patient Population

Patients with genitourinary malignancies enrolling in a clinical trial involving 1 or more oral anticancer therapy were eligible if they had consented but not yet started on 1 of several selected parent clinical trials. When a patient was consenting to 1 of the selected parent clinical trials, the provider would discuss this ancillary trial and assess patient interest. Eligible patients were English-speaking and had adequate internet competency and use, as determined by a 3-point scale eligibility questionnaire. Patients’ level of functioning and ability to perform activities of daily living as well as physical ability were recorded using the Eastern Cooperative Oncology Group (ECOG) Scale of Performance Status and included in the baseline characteristics. Support was not available to develop videos in multiple languages. Patients who were interested and eligible were consented at the same time they were consented to the parent clinical trial. If the provider was unable to collect consent to this ancillary trial on the day of parent trial consent, it was possible to obtain consent and receive registration and randomization status before the initial cycle 1 day 1 clinic visit. The IRB deemed this trial low risk and determined it acceptable for clinical research coordinators trained on the study to consent patients, if delegated by the principal investigator.

### Study Objectives

The primary objective of this trial was to assess the differences in the number of patient-initiated violations in the intervention arm compared with the control arm over 4 treatment cycles. Patient-initiated protocol violations reflect incidents of patient misunderstanding that could potentially be improved upon with the personalized, video-based intervention. Although patient-initiated protocol violations could be measured quantitatively and objectively, the degree to which patient-initiated protocol violations affect the clinical trials process and the rate at which they occur was not known at baseline. This was the rationale for choosing this novel endpoint. Secondary objectives included assessment of patient satisfaction as measured by the Functional Assessment of Chronic Illness Therapy-Treatment Satisfaction-Patient Satisfaction (FACIT-TS-PS) [25], patient perceived stress as measured by the Perceived Stress Scale-10 Item (PSS-10) [26], frequency of webpage use, and number of patient-initiated phone calls to providers.

#### Patient-Initiated Violations

Patient-initiated violations were defined as events that deviated from instructions detailed in the clinical protocol and including inappropriate drug dosing and operational or safety-related events. All instances of violations were recorded by cycle. To give equal weight to the 11 violations, a given violation, no matter how often it occurred within a cycle, counted once per cycle. For example, a patient would have 2 patient-initiated violations if he missed 2 doses (counted as 1 violation) and self-administered 3 doses at the wrong time (counted as 1 violation). We chose to calculate violations as such because each violation reflected 1 episode of patient misunderstanding. Consequently, a patient completing 4 cycles would have a count ranging from 0 to 44 patient-initiated violations.

#### Frequency of Webpage Usage

Postwire allowed for internal tracking of user access, and individual patient and patient designee access was recorded. A designated clinical research coordinator documented individual webpage accession on a weekly basis and inserted the data into the electronic data capture system.

#### Patient Satisfaction and Stress Measures

The FACIT-TS-PS and PSS-10 were collected at each day 1 visit for 6 cycles. The FACIT-TS-PS was used as it is an expansion of the Functional Assessment of Cancer Therapy scale, used specifically in the cancer population. FACIT questionnaires have been robustly validated and address many realms of psychology including physical well-being, social and family well-being, emotional well-being, and functional well-being [27]. The FACIT questionnaire is appropriate for use in patients with any form of cancer, and it has been used and validated in other chronic illnesses, too. The questionnaire can be tailored to include the most relevant questions. Finally, administration time for any 1 assessment is usually less than 15 min.

The PSS-10 predicts both objective biological markers of stress and increased risk for disease among persons with higher perceived stress levels. The PSS-10, which has been externally validated, was chosen because it is one of the most widely used assessments to measure perceived stress and allows patients to appraise what aspects of their lives are stressful [26]. In addition, each question regarding perceived stress is specific to the past month, which is how often most patients on this trial were seen in the clinic. Each question is scored 0 to 4, on a 40-point scale, with scores over 20 representing above average stress. The FACIT-TS-PS contains the following subscales: physician communication, treatment staff communication, technical competence, nurse communication, and confidence and trust. For this study, we did not use the technical competence subscale. On the remaining 4 subscales, there are 23 items, scored 0 to 3, and 1 overall question scored from 0 to 4. This tool is scored on a 73-point scale, with higher scores representing high levels of satisfaction and confidence.

#### Frequently Asked Questions in the Intervention Arm

Questions asked during the filmed patient encounters were retrospectively reviewed and placed into predefined categories by a dedicated clinical research coordinator assigned to this trial. Questions were collected to assess patient clinical trial–related educational needs for future development of educational materials.

#### Patient-Initiated Provider Calls

Standard practice among the DFCI genitourinary clinical trial study team is to document any outgoing or incoming phone calls in the patient electronic medical record. In the event that incoming or outgoing calls were not documented, a clinical research coordinator contacted providers whose patients were participating on this ancillary study for additional phone data. This information was entered into the electronic data capture system unique to this study.

### Statistical Design

To determine an appropriate sample size, the number of cumulative patient-initiated violations over 4 treatment cycles was simulated under a Poisson distribution, accounting for various proportions of patients without any patient-initiated violations. The intervention and control arms were compared with a 1-sided Wilcoxon rank-sum test 3000 times. On the basis of these simulations, the initial sample size was estimated at 75 patients, 25 patients in the control arm and 50 patients in the intervention arm, resulting in 93% power (1-sided alpha .10) to detect a 1:3 rate of patient-initiated violations in the intervention arm to the control arm equivalent to a rate ratio of 33%. This assumed 50% (12.5/25) of the control arm patients would have 1 or more patient-initiated violation or violations. Power was reduced to 80% if fewer patients experienced 1 or more patient-initiated violation or violations (33%).

Given early dropout of patients from their parent clinical trials (causing subsequent removal from this ancillary study), the sample size was expanded from 75 to 99 patients (66 intervention: 33 control) to enable well-powered analyses based on the original hypothesis. Patient dropout was because of our patient population’s aggressive and advanced disease states. A total of 61 out of 89 (69%) patients were enrolled in parent trials related to metastatic cancers of the genitourinary system (Table 1). Given patient replacement was not permitted on this study, the sample size was expanded to enable well-powered analyses based on the original hypothesis.

### Statistical Analysis

On the basis of the statistical design, the primary analysis was to compare the intervention and control arms using the Wilcoxon rank-sum test (1-sided alpha=.10). The primary endpoint was also evaluated as a violation rate (total numbers of violations per cycle), and the comparison between 2 arms was analyzed using a Poisson model. With the Poisson model, patients who dropped off before 4 cycles contributed to the endpoint for as long as they remained in the study. A log-linked model was applied for rate as a function of the predictor variable. By including the log of the treatment cycles into the model, treatment cycles became the denominator of log of the rate of total number of violations. Pearson chi-square was used to estimate the dispersion parameter to account for overdispersion in the model. Standard errors of regression coefficients were adjusted as well. As a sensitivity analysis, exact Wilcoxon rank-sum test adjusting for ties was used to assess whether total number of violations differed by arms in patients who completed all 4 cycles. Descriptive statistics were used to summarize secondary endpoints by arm and cycle.

## Results

### Baseline Patient Characteristics

A total of 99 patients were enrolled from September 2014 to November 2016 (Multimedia Appendix 2). In total, 90% (89/99) of patients were evaluable, of whom 63% (56/89) were in the intervention arm and 37% (33/89) were in the control arm. A total of 74% (66/89) of evaluable patients completed 4 treatment cycles. Reasons for early study discontinuation (23/89, 26%) included disease progression (17/23, 74%), adverse events on primary study therapy (2/23, 9%), withdrawal (1/23, 4%), and unknown (3/23, 13%). In total, 76% (68/89) of patients on this study had prostate cancer, and 24% (21/89) had renal cell cancer in the final analysis. Baseline and disease characteristics are detailed in Table 1.

Most patients had metastatic disease (61/89, 69%), were enrolled in parent trials of hormonal therapy (54/89, 61%), and had ECOG performance scores of 0 (70/89, 79%). The ECOG performance scale is routinely used to assess how patients’ cancers are progressing holistically. A score of 0 means a patient is fully active, and a score of 5 means death [28]. In total, 64% (57/89) were *extremely confident* in their use of the internet, whereas the remaining patients (32/89, 36%) were *somewhat confident* in their use of the internet.

### Patient-Initiated Violations

Of the 89 patients analyzed, 71% (40/56) of patients in the intervention arm had 1 or more patient-initiated violations compared with 70% (23/33) of patients in the control arm. Table 2 describes the total violations.

For the intervention arm, the range of violations was 0 to 11, with a median of 1 in patients who completed greater than or equal to 4 cycles. For the control arm, the range of violations was 0 to 7, with a median of 1 in patients who completed greater than or equal to 4 cycles. For all patients who completed greater than or equal to 4 cycles, the range of violations was 0 to 11, with a median of 1. In total, 71% (63/89) had 1 or more violations over the course of 4 cycles.

Improper dosing (, 2/89, 2%), improper concomitant medication administration (5/89, 6%), and wrong doses (5/89, 6%) were among the patient-initiated violations. The most common patient-initiated violations were procedural in nature and included failing to return unused medication for pharmacy drug accountability (16/89, 18%), failing to return medication diaries (10/89, 11%), and incomplete medication diaries (6/89, 7%). There was no difference in the total number of patient-initiated violations across 4 cycles between arms (estimate=−0.0939, 95% CI −0.6295 to 0.4418, *P* value=.73). As a sensitivity analysis, the total number of patient-initiated violations was summarized for patients who completed 4 cycles of the intervention. There was no difference in total patient-initiated violations between arms (*P*=.92) in the 66 patients who completed greater than or equal to 4 cycles.

**Table 1:** Baseline patient characteristics.

Analysis population baseline characteristics			Arm 1 (N=56), n (%)	Arm 2 (N=33), n (%)	All (N=89), n (%)
**Disease stage**					
	Metastatic		39 (70)	22 (67)	61 (69)
	Nonmetastatic		17 (30)	11 (33)	28 (32)
**Disease type**					
	Renal cell carcinoma		12 (21)	9 (27)	21 (24)
	Prostate adenocarcinoma		44 (79)	24 (73)	68 (76)
**Parent protocol type**					
	Unknown		1 (2)	2 (6)	3 (3)
	Combination therapy		10 (18)	5 (15)	15 (17)
	Hormone therapy		35 (63)	19 (58)	54 (61)
	Targeted therapy		10 (18)	7 (21)	17 (19)
**Eastern Cooperative Oncology Group**					
	0		45 (80)	25 (76)	70 (79)
	1		10 (18)	8 (24)	18 (20)
	2		1 (2)	—^a^	1 (1)
**Internet use confidence scale**					
	Extremely confident		38 (68)	19 (58)	57 (64)
	Somewhat confident		18 (32)	14 (42)	32 (36)
**Frequency of internet access**					
	1 time a week		1 (2)	1 (3)	2 (2)
	2 times a week		1 (2)	1 (3)	2 (2)
	5 or more times a week		54 (96)	31 (94)	85 (96)
**Number of times per week internet is accessed**					
	1 time a week		1 (2)	1 (3)	2 (2)
	2 times a week		—	1 (3)	1 (1)
	3 times a week		—	1 (3)	1 (1)
	4 times a week		1 (2)	—	1 (1)
	5 or more times a week		54 (96)	30 (91)	84 (94)
	All		56 (100)	33 (100)	89 (100)
**Baseline characteristics in patients who completed greater than or equal to 4 cycles**					
	**Parent protocol type**					
		Unknown	1 (2)	1 (4)	2 (3)
		Combination therapy	5 (12)	1 (4)	6 (9)
		Hormone therapy	34 (79)	16 (70)	50 (76)
		Targeted therapy	3 (7)	5 (22)	8 (12)
	**Disease stage**				
		Metastatic	26 (60)	12 (52)	38 (58)
		Nonmetastatic	17 (41)	11 (48)	28 (42)
		All	43 (100)	23 (100)	66 (100)

^a^Not applicable.

**Table 2:** Summary of overall frequency and type of patient-initiated violations by arm.

Patient-initiated violations by arm			Arm 1 (N=56), n (%)	Arm 2 (N=33), n (%)	All (N=89), n (%)
**Total patient-initiated violations in the analysis population**					
	0		16 (28)	10 (30)	26 (29)
	1		15 (26)	9 (27)	24 (27)
	2		12 (21)	4 (12)	16 (18)
	3		9 (16)	4 (12)	13 (14)
	4		1 (1)	3 (9)	4 (4)
	5		—^a^	1 (3)	1 (1)
	6		1 (1)	1 (3)	2 (2)
	7		1 (1)	1 (3)	2 (2)
	11		1 (1)	—	1 (1)
	All		56 (100)	33 (100)	89 (100)
**Summary of type of patient-initiated violations in the analysis population**					
	**Incomplete medication diary**				
		1	5 (8)	1 (3)	6 (6)
		3	2 (3)	2 (6)	4 (4)
		4	—	1 (3)	1 (1)
		Total	7 (12)	4 (12)	11 (12)
	**Failure to return medication diary to visit**				
		1	4 (7)	1 (3)	5 (5)
		3	2 (3)	2 (6)	4 (4)
		4	—	1 (3)	1 (1)
		Total	6 (10)	4 (12)	10 (11)
	**Failure to return leftover medication to visit**				
		1	3 (5)	2 (6)	5 (5)
		3	8 (14)	2 (6)	10 (11)
		4	—	1 (3)	1 (1)
		Total	11 (19)	5 (15)	16 (18)
	**Failure to notify study team of interim cycle adverse event**				
		1	3 (5)	2 (6)	5 (5)
		3	5 (8)	—	5 (5)
		4	2 (3)	—	2 (2)
		Total	10 (17)	2 (6)	12 (13)
	**Failure to notify study team of interim emergency department visit**				
		1	1 (1)	—	1 (1)
		3	1 (1)	—	1 (1)
		Total	2 (3)	0 (0)	2 (2)
	**Number of improper doses**				
		6	—	1 (3)	1 (1)
		12	1 (1)	—	1 (1)
		Total	1 (1)	1 (3)	2 (2)
	**Number of improper self con-medication administration**				
		1	3 (5)	—	3 (3)
		3	—	1 (3)	1 (1)
		12	—	1 (3)	1 (1)
		Total	3 (5)	2 (6)	5 (5)
	**Number of missed appointments**				
		1	—	1 (3)	1 (1)
		5	1 (1)	—	1 (1)
		12	—	1 (3)	1 (1)
		Total	1 (1)	2 (6)	3 (3)
	**Number of missed doses**				
		1	3 (5)	2 (6)	5 (5)
		3	5 (8)	3 (9)	8 (9)
		6	3 (5)	—	3 (3)
		15	1 (1)	—	1 (1)
		Total	12 (21)	5 (15)	17 (19)
	**Number of wrong doses**				
		1	2 (3)	—	2 (2)
		3	2 (3)	1 (3)	3 (3)
		Total	4 (7)	1 (3)	5 (5)
	**Number of doses self-administered at the wrong time**				
		1	—	1 (3)	1 (1)
		3	2 (3)	2 (6)	4 (4)
		4	—	1 (3)	1 (1)
		Total	2 (3)	4 (12)	6 (6)

^a^Not applicable.

**Table 3:** Webpage accession by cycle for patients in the intervention arm.

Cycle number and number of times webpage accessed		n (%)
**1**
	0	17 (30)
	1	13 (23)
	2	17 (30)
	3	3 (5)
	4	2 (3)
	5	2 (3)
	6	1 (1)
	14	1 (1)
	All	56 (100)
**2**		
	0	29 (53)
	1	15 (27)
	2	6 (11)
	3	3 (5)
	4	1 (1)
	All	54 (100)
**3**		
	0	30 (66)
	1	13 (28)
	2	2 (4)
	All	45 (100)
**4**		
	0	26 (60)
	1	11 (25)
	2	3 (7)
	3	2 (4)
	4	1 (2)
	All	43 (100)

### Frequency of Webpage Usage

Table 3 describes webpage accession by patients and their designees.

In total, 70% (39/56) of patients assigned to the intervention arm (including 9 designees) accessed the video-based, personalized webpage and its subcontents during cycle 1, 45% (25/56) during cycle 2, 27% (15/56) during cycle 3, and 25% (14/56) during cycle 4. During the 2 follow-up cycles, 5 patients accessed the webpage during cycle 5, and 1 patient accessed the webpage during cycle 6.

### Patient Satisfaction and Stress Measures

The median baseline satisfaction score for the intervention arm was 72 and 73 for the control arm (Table 4). Following baseline, the median satisfaction score for both arms at each time point was 73. The median baseline stress level for the intervention arm was 10 and 13 for the control arm (Table 5). Median stress levels for the intervention arm at cycles 2 to 6 were 9, 8, 8, 9, and 7, respectively. Median stress levels for the control arm at cycles 2 to 6 were 11.5, 10.5, 10, 11, and 9.5, respectively. Median stress score for both arms at the end of study (following 6 cycles) was 7.

**Table 4:** Patient satisfaction scores.

Cycle and arm		Minimum	Median	Maximum
**1**			
	1 (N=37)	53	72	73
	2 (N=26)	55	73	73
	All (N=63)	53	72	73
**2**				
	1 (N=50)	54	73	73
	2 (N=29)	66	73	73
	All (N=79)	54	73	73
**3**				
	1 (N=46)	29	73	73
	2 (N=26)	68	73	73
	All (N=72)	29	73	73
**4**				
	1 (N=34)	60	73	73
	2 (N=22)	55	73	73
	All (N=56)	55	73	73
**5**				
	1 (N=38)	48	73	73
	2 (N=18)	67	73	73
	All (N=56)	48	73	73
**6**				
	1 (N=37)	49	73	73
	2 (N=18)	68	73	73
	All (N=55)	49	73	73
**End of study**				
	1 (N=39)	53	73	73
	2 (N=19)	72	73	73
	All (N=58)	53	73	73

**Table 5:** Patient perceived stress scores.

Cycle and arm		Minimum	Median	Maximum
**1**				
	1 (N=54)	0	10	25
	2 (N=31)	0	13	28
	All (N=85)	0	11	28
**2**				
	1 (N=53)	0	9	24
	2 (N=30)	0	11.5	21
	All (N-83)	0	11	24
**3**				
	1 (N=40)	0	8	27
	2 (N=24)	0	10.5	20
	All (N=64)	0	9	27
**4**				
	1 (N=47)	0	8	25
	2 (N=27)	0	10	23
	All (N=74)	0	8.5	25
**5**				
	1 (N=41)	0	9	25
	2 (N=21)	0	11	22
	All (N=62)	0	9.5	25
**6**				
	1 (N=41)	0	7	25
	2 (N=20)	0	9.5	22
	All (N=61)	0	8	25
**End of study**				
	1 (N=43)	0	7	24
	2 (N=21)	0	7	24
	All (N=64)	0	7	24

**Table 6:** Number of questions asked during the video encounters, according to type of question and cycle.

Cycle and question type		Median number of questions asked by patient (interquartile range)	Maximum
**1 (n=56)**				
	Drug administration	2 (1-4)	10
	Drug handling	0 (0-1)	4
	Research team contact	0.5 (0-1)	3
	Scheduling	1 (0.5-2)	8
	Side effects	1 (0-3.5)	8
	Webpage	0 (0-0)	2
**2 (n=52)**				
	Drug administration	0 (0-1)	7
	Drug handling	0 (0-0)	4
	Research team contact	0 (0-0)	6
	Scheduling	0 (0-1)	3
	Side effects	0 (0-1)	5
	Webpage	0 (0-0)	2
**3 (n=44)**				
	Drug administration	0 (0-2)	4
	Drug handling	0 (0-0)	5
	Research team contact	0 (0-0)	1
	Scheduling	0 (0-1)	7
	Side effects	1 (0-2)	7
	Webpage	0 (0-0)	1
**4 (n=43)^a^**				
	Drug administration	0 (0-1)	4
	Drug handling	0 (0-0)	4
	Research team contact	0 (0-0)	1
	Scheduling	0 (0-1)	3
	Side effects	1 (0-2)	7
	Webpage	0 (0-0)	2

^a^Data missing on 1 patient.

### Frequently Asked Questions in the Intervention Arm

The most frequently asked questions by patients during the video encounters were tabulated for individuals in the intervention arm. Most questions asked were related to drug administration, drug side effects, and scheduling (Table 6).

### Patient-Initiated Provider Calls

Overall, 21% (19/89) of patients in this study called an oncologist or nurse practitioner (Table 7) at least once (range 1-5), and 73% (65/89) of patients on study called a research nurse at least once (range 1-12).

**Table 7:** Patient-initiated calls to providers.

Cycle and call type			Arm 1, n (%)	Arm 2, n (%)
**1**			
	**Calls to MD^a^** **or NP^b^**		
		1	2 (4)	2 (6)
	**Calls to RN^c^**		
		1	12 (21)	6 18)
		2	7 (13)	2 (6)
		3	1 (2)	4 (12)
**2**				
	**Calls to MD or NP**			
		1	4 (8)	1 (3)
	**Calls to RN**			
		1	10 (20)	9 (30)
		2	5 (10)	2 (7)
		3	0 (0)	1 (3)
		4	3 (6)	0 (0)
		5	0 (0)	2 (7)
**3**				
	**Calls to MD or NP**			
		1	1 (2)	0 (0)
		2	1 (2)	0 (0)
	**Calls to RN**			
		1	11 (24)	4 (15)
		2	3 (7)	3 (11)
		3	1 (2)	0 (0)
		4	2 (4)	0 (0)
**4**				
	**Calls to MD or NP**			
		1	1 (2)	0 (0)
	**Calls to RN**			
		1	8 (19)	3 (13)
		2	1 (2)	0 (0)

^a^Medical Doctor.

^b^Nurse Practitioner.

^c^Registered Nurse.

## Discussion

### Principal Findings

In this study, we evaluate a video-based, personalized webpage as a complement to routine clinical trial patient education. Our trial did not prove that the video-based, personalized webpage intervention reduced patient-initiated violations. This result might in part be related to the low total number of patient-initiated violations in both arms. Nearly one-third of patients had no violations, and 27% (24/89) had only 1 violation over the course of 4 cycles. We suspect that given the sophistication needed to seek care at an urban tertiary cancer center and enroll in, not 1 but 2 clinical trials [29], our patient population was perhaps more engaged in their cancer care than the general cancer population, contributing to the low number of patient-initiated violations observed in this trial. Such an intervention might be of more use in a less engaged patient population to aid in adherence of oral anticancer therapy prescribed in a standard of care setting.

Due to the exclusion criteria of the parent clinical trials and the lack of resources necessary to create materials for non-English-speaking patients, only English-speaking patients were included in this study. Research has touched upon the lack of minority representation in clinical trials. Video education has been shown to increase non-English-speaking patients’ understanding of consent information compared with oral education and home visits and has helped with recruiting and retaining non-English speaking patients in clinical trials [30]. This video-based, personalized webpage might be of use in the recruitment of a greater number of minority patients in oncology clinical trials.

The FACIT-TS-PS and PSS-10 results show that patients were satisfied with their research teams and stress levels were low in both arms. We suspect that this is because patients who remained on study were deriving clinical benefit from their parent clinical trials, thus impacting their satisfaction and stress levels. Though generally patients were satisfied with their care teams, experienced low levels of stress, and were relatively compliant regarding patient-initiated violations, there remain opportunities for continued improvement of communication and education in the clinical trials process [8,19].

On the basis of patient feedback in the form of emails to the study team, patient caregivers appeared to derive benefit from the intervention. Future work can assess the effect of similar interventions on patient family members or caregivers as a means of supporting the patient and improving the clinical trial experience. Future research might also consider enabling webpage communication functions, allowing researchers to assess the utility of real-time, Web-based modes of communication over standard methods [31]. Video recording by use of iPads required trained personnel in the exam room for each encounter. This approach is not well suited for environments with limited staffing resources. Future studies should consider the use of remote video-recording to eliminate this need. Further development of this intervention should also explore mobile phone accessibility.

### Limitations

To objectively quantify the impact of the video-based, personalized webpage, we selected an innovative primary endpoint for this study: patient-initiated violations. Developing the primary endpoint was challenging given the lack of clinical studies of this type. We chose patient-initiated violations because this endpoint was quantifiable and clinically meaningful. Furthermore, we thought that this novel primary endpoint would serve as a surrogate of patient knowledge of trial medication adherence, administration, and overall clinical trial procedures. Defining the null and alternative hypotheses at the onset of the study was also difficult, as there are no published examples of patient-initiated violations in similar patients. An alternative endpoint that has been used in other studies of video-based education delivery includes patient-reported outcomes of knowledge, attitude, and preparation for making decisions about clinical trials [18]. Such an endpoint should be considered in future research assessing this intervention.

The intervention might have broader impact in a less resourced setting or community practice to enhance clinical trial procedures and patient education as well as in settings outside the realm of clinical trials. This trial, which was conducted at a single institution with extensive experience in clinical trial operations, required patients to have access to the internet and some degree of confidence in internet use. These enrollment criteria might have biased the patient population to those with the most potential for understanding clinical trial procedures and excluded those who could have experienced the greatest benefit.

### Comparison With Prior Work

Strategies to incorporate Web-based and mobile communication on clinical trials are underway, such as the Preparatory Education About Clinical Trials, a Web-based and interactive computer program delivering educational content to patients considering clinical trials, and a Web-based prostate cancer treatment decision aid assessing treatment satisfaction, decisional regret, and quality of life [18,21,32]. Advances in technology, such as video-based interventions and mobile apps, are expected to impact the delivery of oncology care over the next decade [33]. Advantages of e-technology (computer-assisted interventions and mobile phone apps) in clinical trials include improved efficiency, cost reduction, and fostering research and development [32]. These interventions have the potential to enhance patient understanding, improve patient enrollment, and streamline clinic operations [18].

### Conclusions

As the population in general becomes more skilled with technology, we believe this type of intervention will be useful for patients and providers of varying disciplines. Although the primary endpoint of reduced patient-initiated violations over 4 cycles of treatment in the intervention arm was not met, this study demonstrates the feasibility of providing patients and caregivers with instant, at-home access to the discussions that take place between patients and their care teams. There was no difference between arms at the end of 4 cycles or following 2 cycles of follow-up. Nevertheless, our video-based personalized webpage offered patients an alternative to impersonal, lengthy, and written information as is currently provided in informed consent documents. Our intervention allowed patients to store, share, and access complex discussions related to their cancer care repeatedly over extended periods. With the ever-increasing role of technology in health care, the potential of a video-based, personalized webpage in oncology care includes optimization of oral anticancer therapy adherence, management of drug side effects, increase in patient safety, and improved trial operational quality and efficacy in a sustainable and translatable way that could benefit patients and providers to come.

## Appendix

Multimedia Appendix 1 Your clinical trial research nurse explains how to take your doses of abiraterone acetate and prednisone each day.

Multimedia Appendix 2 Consolidated Standards of Reporting Trials (CONSORT) diagram.

Multimedia Appendix 3 CONSORT-EHEALTH checklist (V 1.6.1).

## Abbreviations

DFCI: Dana-Farber Cancer Institute
ECOG: Eastern Cooperative Oncology Group
FACIT: Functional Assessment of Chronic Illness Therapy
FACIT-TS-PS: Functional Assessment of Chronic Illness Therapy-Treatment Satisfaction-Patient Satisfaction
FDA: Food and Drug Administration
IRB: institutional review board
PSS-10: Perceived Stress Scale-10 Item
