# Supplementary figures and images for: Evaluating a Video-Based, Personalized Webpage in Genitourinary Oncology Clinical Trials: A Phase 2 Randomized Trial
Source: J Med Internet Res. 2019 May 2;21(5):e12044. doi: 10.2196/12044 (PMC6538310; doi:10.2196/12044)

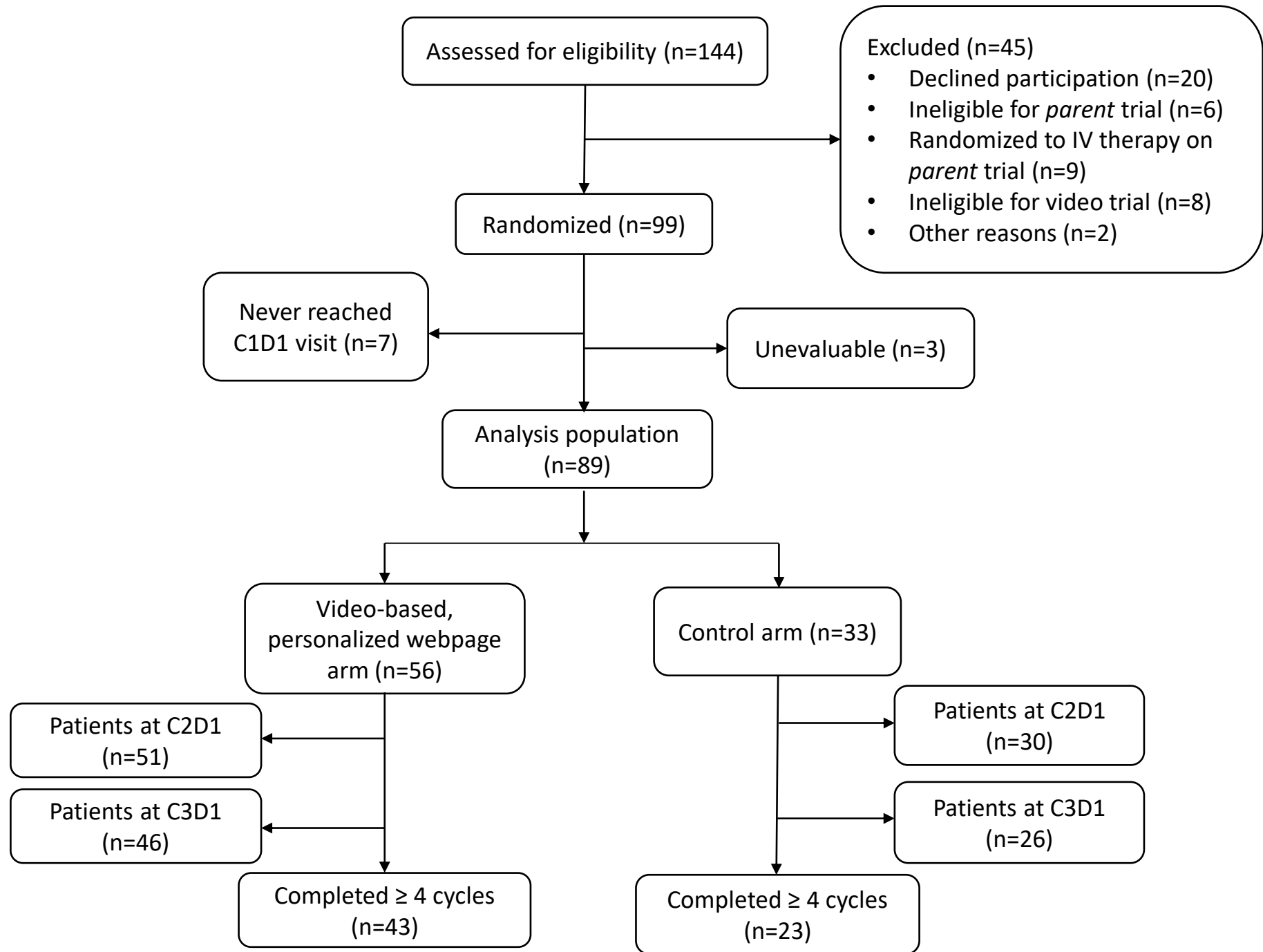

Supplement: Multimedia Appendix 2 [file jmir_v21i5e12044_app2.pdf]
